# Utility of Small Bowel Capsule Endoscopy and Leucine-Rich Alpha-2-Glycoprotein in Pediatric Crohn’s Disease Management

**DOI:** 10.7759/cureus.99368

**Published:** 2025-12-16

**Authors:** Satoshi Ukai, Shun Watanabe, Ayako Furuya, Tomomitsu Sado, Shingo Kurasawa, Atsuhiro Hirayama, Sawako Kato, Yoshiko Nakayama

**Affiliations:** 1 Pediatrics, Shinshu University School of Medicine, Matsumoto, JPN; 2 Gastroenterology, Shinshu University School of Medicine, Matsumoto, JPN

**Keywords:** capsule endoscopy crohn’s disease activity index, crohn's disease, leucine-rich alpha-2 glycoprotein, lewis score, pediatric crohn's disease, pediatric crohn’s disease activity index, small bowel capsule endoscopy, treat to target

## Abstract

Introduction

Mucosal remission is a key treatment goal for Crohn’s disease (CD). Small-bowel capsule endoscopy (SBCE) is a minimally invasive modality for evaluating small-bowel mucosa; however, standardized criteria for mucosal remission have not yet been established. This study aimed to investigate the correlation and comparative sensitivity of the Lewis score (LS) and Capsule Endoscopy Crohn’s Disease Activity Index (CECDAI) in assessing small-bowel inflammation in pediatric Crohn’s disease (CD) and to evaluate biomarkers as predictors of small bowel mucosal remission.

Methods

This cohort study included pediatric patients with CD at a single center in Japan. Demographics, clinical symptoms, serum C-reactive protein (CRP) levels, erythrocyte sedimentation rate (ESR), and leucine-rich alpha-2 glycoprotein (LRG) levels were evaluated. SBCE findings were scored using LS and CECDAI.

Results

Among 26 patients, 54 SBCEs were performed, as some patients underwent multiple examinations. Median LS and CECDAI scores were 403 and 12, respectively. LS and CECDAI showed positive correlation (ρ = 0.82, p < 0.001). LS values of 135 and 790 corresponded to CECDAI of 7.1 and 16.5, respectively. Among 32 clinical remission cases, 24 had LS ≥ 135, while 17 had CECDAI ≥ 7.1. CRP, ESR and LRG correlated with SBCE findings, with LRG showing the strongest association (ρ = 0.74, p = 0.01). LRG < 13.4 µg/mL predicted small bowel mucosal remission.

Conclusions

LS and CECDAI showed a strong correlation, supporting their utility in evaluating small bowel inflammation in pediatric Crohn’s disease. LS may have a tendency to detect milder or subclinical inflammatory changes, although the magnitude of this difference should be interpreted cautiously. In addition, LRG < 13.4 µg/mL strongly predicted mucosal remission, and these findings, though limited by sample size, suggest that LRG combined with clinical indices could serve as a noninvasive adjunct for timing SBCE.

## Introduction

Crohn’s disease (CD) is a granulomatous inflammatory disorder of the gastrointestinal tract. In recent years, therapeutic goals have shifted from clinical remission (CR) to mucosal remission (MR) [1]. Recent epidemiological studies have demonstrated that the incidence and prevalence of pediatric inflammatory bowel disease (IBD), including CD, are increasing worldwide. Pediatric patients with CD in Japan have been reported to show a lower prevalence of colonic disease and a higher prevalence of ileocolonic involvement than those in Europe [2]. Consequently, mucosal lesions in the small bowel may be overlooked when relying solely on colonoscopy for mucosal evaluation [3]. Small-bowel capsule endoscopy (SBCE) has been shown to be useful for assessing the small bowel mucosa [4-6]. However, SBCE has some limitations, as it only identifies mucosal lesions, and standardized remission thresholds for children have not yet been established. In addition, capsule retention occurs in 1.64% of pediatric patients with CD [7].

Among the scoring systems based on SBCE findings, the Lewis score (LS) and the Capsule Endoscopy Crohn’s Disease Activity Index (CECDAI) are commonly used [8,9]. However, no standardized criteria have been established to define MR in patients with CD using SBCE [10]. Although previous studies in adults have demonstrated a positive correlation between LS and CECDAI [11-13], limited data are available on the relationship between these two indices in pediatric populations.

However, these endoscopic indices require invasive assessment, underscoring the need for reliable non-invasive biomarkers. Leucine-rich α₂-glycoprotein (LRG), an inflammation-induced glycoprotein produced by neutrophils and intestinal epithelial cells, has recently emerged as a promising biomarker in IBD, showing closer correlation with mucosal inflammation than C-reactive protein (CRP) [14,15].

This study’s primary objective was to clarify the correlation between the LS and CECDAI in pediatric patients with CD. Secondary objectives were to examine the relationships between endoscopic scores and biomarkers, and to evaluate the predictive ability of biomarkers for MR in the small bowel.

## Materials and methods

Ethics statement

This study was conducted in accordance with the Declaration of Helsinki and approved by the Ethics Committee of Shinshu University School of Medicine (approval number: 6211). The data were collected between June and December 2024.

Patients

This study included patients diagnosed with CD who visited the Shinshu University Hospital between January 2011 and May 2024. The inclusion criterion was all pediatric patients aged < 18 y who had undergone SBCE. The exclusion criteria were: diagnosis of colonic-type CD, age ≥ 18 years at the time of SBCE, suspected structural small bowel stenosis based on imaging, or SBCE performed under inappropriate conditions such that mucosal evaluation was not feasible. No formal sample size calculation was performed, as this retrospective study included all consecutive eligible patients who underwent SBCE during the study period.

Study design

The collected data included age, sex, age at diagnosis, and disease location, classified according to the Paris Classification [16]. The clinical status at the time of SBCE, including age and Pediatric Crohn’s Disease Activity Index (PCDAI), was also evaluated. The PCDAI is a validated clinical scoring system used to assess disease activity in children with CD. It incorporates multiple domains, including clinical symptoms (such as abdominal pain, stool frequency, and general well-being), physical examination findings, growth parameters, and laboratory markers of inflammation. Scores range from 0 to 100, with higher scores indicating more severe disease activity [17]. The PCDAI score of < 10 was defined as CR.

SBCE was performed using a PillCam SB2 or SB3 (Medtronic, Minneapolis, MN, USA). The PillCam SB2, introduced in 2007, captures images at a fixed frame rate of two frames per second. In contrast, the PillCam SB3, released in 2013, offers improved image resolution and employs an adaptive frame rate technology (two to six frames per second), allowing automatic adjustment according to capsule movement speed. SBCE was conducted for one of the following clinical purposes: evaluation of disease extent, assessment of therapeutic response, or surveillance. Patency capsules were used as needed before SBCE, and intestinal patency was confirmed when the capsule was excreted intact within approximately 30 hours. Small-bowel transit time (SBTT) and whether the cecum was reached within the examination period were assessed. SBTT was defined as the time interval between the first duodenal image and the first cecal image. In patients who had difficulty swallowing the capsule, we introduced the capsule into the duodenum under esophagogastroduodenoscopic guidance. Inflammatory changes were evaluated using the LS and CECDAI retrospectively. LS was calculated using Rapid 8.3 software, and CECDAI was calculated manually. For SBCEs that did not reach the cecum, the LS and CECDAI were calculated for the observed portion up to the final frame. The LS and CECDAI were independently scored by a single reviewer (S.U.), who was blinded to the clinical information.

We evaluated CRP levels, erythrocyte sedimentation rate (ESR), and LRG as biomarkers. LRG is a 50-kDa glycoprotein characterized by repetitive leucine-rich motifs. It can be measured in blood samples and reflects inflammatory signaling mediated by proinflammatory cytokines such as TNF-α, IL-1β, IL-6, and IL-22. LRG is predominantly produced by neutrophils, macrophages, intestinal epithelial cells, and hepatocytes in response to these cytokines and is increasingly used as a biomarker for IBD [14,15]. In Japan, serum LRG testing became reimbursable under the national health insurance system in 2020, and all LRG measurements in this study were conducted within this insurance coverage. Because fecal calprotectin (FC) and LRG cannot be reimbursed simultaneously under the current insurance regulations, FC data were not consistently obtainable during the study period. Furthermore, LRG has been reported to more sensitively reflect small-bowel inflammation in CD compared with FC. For these reasons, serum LRG was selected as the biomarker for analysis in this study. Serum LRG was measured using a commercially available assay (Nanopia LRG, Sekisui Medical, Tokyo, Japan), which employs a latex agglutination method with an anti-human LRG mouse monoclonal antibody. According to the manufacturer's reference values, the upper limit of normal for serum LRG is 16 µg/mL. Biomarkers evaluated within two months before or after the SBCE were included in the analysis. However, if treatment was modified during this period, only biomarker data obtained under the same treatment regimen as at the time of SBCE were used.

LS was developed to quantify small-bowel inflammation by assessing three parameters: villous edema, ulceration, and strictures. The SBTT was divided into three equal segments; villous edema and ulceration were scored for each segment, and the stricture score was added to the most severely affected segment. Mucosal remission is defined as LS < 135; mild inflammation as 135 ≤ LS < 790; and moderate to severe inflammation as LS ≥ 790 [8].

The CECDAI is a scoring system specific to SBCE for evaluating CD activity based on inflammation, disease extent, and strictures. The SBTT was divided into two segments, and all three parameters were scored. The total CECDAI score is the sum of the scores from both segments [9]. Although no cut-off was defined in the original report, higher scores indicated more severe inflammation.

The primary outcome was the correlation between LS and CECDAI scores. Secondary outcomes included the relationship between SBCE findings and PCDAI or biomarkers at the time of SBCE.

Statistical analysis

Continuous variables are expressed as medians [interquartile range (IQR)], and categorical variables are expressed as percentages. Normality was assessed using the Shapiro-Wilk test. Because some patients underwent multiple SBCE examinations, we assessed the potential non-independence of repeated measures before conducting the correlation analysis. A linear mixed-effects model with patient ID as a random intercept was used to estimate the intraclass correlation coefficient (ICC). The ICC was 0.13, indicating minimal within-patient clustering. Therefore, Spearman’s rank correlation coefficient was used to evaluate the association between LS and CECDAI. The CECDAI values corresponding to LS scores of 135 and 790 were estimated using linear regression. Categorical variables were compared using Fisher’s exact test, and continuous variables were compared using the t-test, Mann-Whitney U test, or Kruskal-Wallis test, as appropriate. Receiver operating characteristic (ROC) curve analysis was performed to assess the predictive value of the biomarkers for MR based on SBCE. The optimal cutoff values were determined using the Youden index. Statistical significance was defined as a two-sided p-value of < 0.05. Statistical analyses were conducted using the EZR software (Jichi Medical University, Saitama, Japan).

## Results

**Figure 1:**
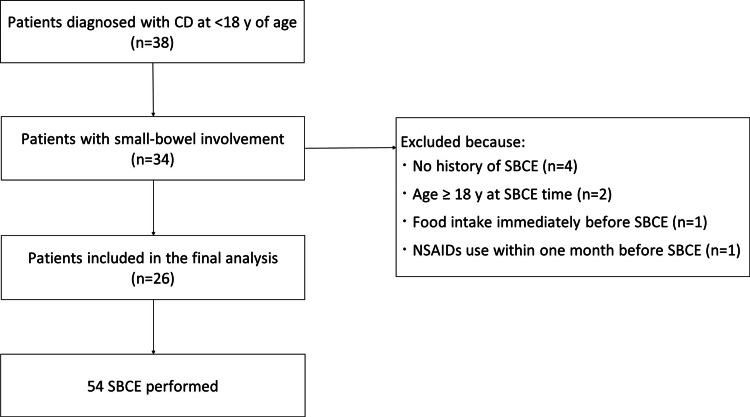
Flowchart of patients included Of 38 patients diagnosed with CD at < 18 y, 34 with small-bowel involvement were assessed. After excluding 8 patients based on predefined criteria, 26 patients were included in the final analysis, in whom a total of 54 SBCE were performed. CD, Crohn’s disease. SBCE: small-bowel capsule endoscopy.

Of the 38 patients diagnosed with CD at < 18 y of age, 34 with small-bowel involvement were initially considered. Eight patients were excluded for the following reasons: no history of SBCE (n = 4), age ≥ 18 y at the time of SBCE (n = 2), food intake immediately before SBCE (n = 1), or NSAIDs use within one month before SBCE (n = 1). A total of 26 patients were included in the final analysis (Figure 1).

Nineteen patients were male, and the median age at diagnosis was 13 y (IQR 11-14 y). Disease classification based on the Paris classification identified two patients as L1, 24 as L3, 21 as L4a, and 17 as L4b. During the follow-up, 54 SBCE were performed. The median age at the time of SBCE was 13 y (IQR 12-16 y). Among the 53 SBCEs for which the PCDAI was available, 32 were classified as being in CR (PCDAI < 10). The median SBTT was 234 min (IQR 173-329), and the cecum was not reached in 6 cases (Table 1).

**Table 1:** Characteristics of patients and conditions at the SBCE Data are expressed as a number (percentage) or median (interquartile range). Age was subdivided into A1a (< 10 y), A1b (10 – 17 y), A2 (17 – 40 y), and A3 (> 40 y), reflecting disease onset timing. Disease locations included L1 (distal 1/3 ileum), L2 (colonic), L3 (ileocolonic), L4a (upper disease proximal to the Ligament of Treitz), and L4b (upper disease distal to the ligament of Treitz and proximal to the distal 1/3 ileum). The Ligament of Treitz was identified by tracking capsule movement using the sensor array system. Disease behavior was classified as B1 (nonstricturing, non-penetrating), B2 (stricturing), B3 (penetrating), or B2B3 (both features). SBCE: small-bowel capsule endoscopy; PCDAI: Pediatric Crohn’s Disease Activity Index; LS: Lewis score. CECDAI: Capsule Endoscopy Crohn’s Disease Activity Index.

Parameter	Value	
Number of patients	26	
Sex(male/female)	19/7	(73%/27%)
Age at diagnosis [median (IQR)] (years)	13	(11 - 14)
Paris classification		
A1a	6	(23%)
A1b	19	(73%)
A2	1	(4%)
A3	0	(0%)
L1	2	(8%)
L2	0	(0%)
L3	24	(92%)
L4a	21	(81%)
L4b	17	(65%)
B1	26	(100%)
B2	0	(0%)
B3	0	(0%)
Number of SBCE	54	
Age at SBCE [median (IQR)] (years)	13	(12 - 16)
Disease duration [median (IQR)] (month)	19	(7 - 41)
PCDAI [median (IQR)]	5	(0 - 15)
< 10[n]	32	(59%)
≥ 10[n]	21	(39%)
Small bowel transit time [median (IQR)] (min)	234	(173 - 329)
CRP [median (IQR)] (mg/L)	0.8	(0.2 - 3.7)
ESR [median (IQR)] (mm/h)	9	(4 - 19)
LRG [median (IQR)] (µg/mL)	16.5	(11.3 - 28.6)
LS [median (IQR)]	403	(233 - 562)
< 135[n]	8	(15%)
135 - < 790 [n]	41	(76%)
790 - [n]	5	(9%)
CECDAI [median (IQR)]	12	(6 - 15)
< 7.1 [n]	16	(30%)
7.1 - < 16.5 [n]	29	(54%)
16.5 - [n]	9	(17%)

**Figure 2:**
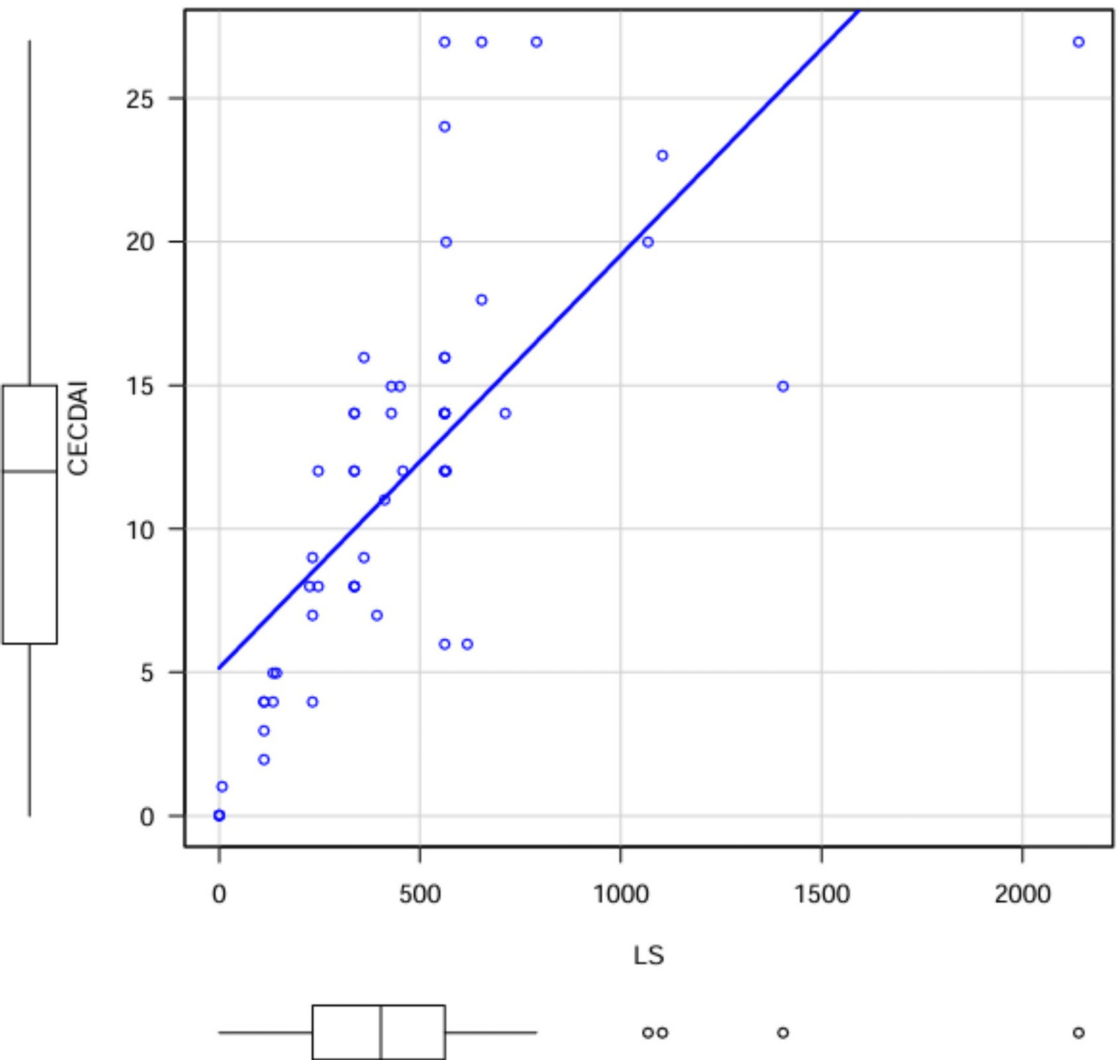
Correlation of LS and CECDAI The median LS was 403 (IQR 233 - 562); the median CECDAI was 12 (IQR 6 - 15) for the 54 SBCE cases. Spearman's rank correlation coefficient showed a positive correlation between LS and CECDAI scores. (ρ = 0.82, p < 0.001). In the linear regression analysis, LS = 135, 790 corresponded to 7.1, 16.5 in CECDAI. SBCE: small-bowel capsule endoscopy; LS: Lewis score; CECDAI: Capsule Endoscopy Crohn’s Disease Activity Index.

The median LS was 403 (IQR, 233 - 562), and the median CECDAI score was 12 (IQR, 6 - 15). The LS was < 135 in eight cases, 135 - 790 in 41 cases, and ≥ 790 in five cases. LS and CECDAI were strongly positively correlated (ρ = 0.82, p < 0.001). Linear regression indicated that an LS of 135 and 790 corresponded to a CECDAI of 7.1 and 16.5, respectively. CECDAI < 7.1 was found in 16 cases, 7.1 - < 16.5 in 29 cases, and ≥ 16.5 in nine cases (Table 1, Figure 2).

PCDAI and LS were positively correlated (ρ = 0.47, p < 0.001). Among the 32 SBCEs with PCDAI < 10, only eight showed LS < 135, while 24 had LS ≥ 135. All 21 SBCEs with PCDAI ≥ 10 had LS ≥ 135. When categorized into four groups using PCDAI < 10 and LS < 135 as cut-offs, a significant difference was found using Fisher’s exact test (Table 2). Patients were then grouped into MR (PCDAI < 10, LS < 135), CR (PCDAI < 10, LS ≥ 135), and flare (PCDAI ≥ 10, LS ≥ 135) groups. Demographic data, biomarkers, and the number of segments in which the combined villous edema and ulceration subscores, components of the LS, were ≥ 135 were compared across these groups. Comparisons among the three groups revealed significant differences in CRP, ESR, and the number of segments with LS ≥ 135. Although LRG was available in only two MR, two CR, and six flare cases, no significant difference was found (Table 3). Bonferroni post hoc tests showed significant differences between MR and CR for CRP (p = 0.025) and the number of LS ≥ 135 segments (p < 0.001); between MR and flare for CRP (p = 0.002), ESR (p < 0.001), and LS ≥ 135 segments (p < 0.001); and between CR and flare for CRP (p = 0.015) and ESR (p = 0.004). Similarly, PCDAI and CECDAI were positively correlated (ρ = 0.57, p < 0.001). Among the 32 cases with PCDAI < 10, 15 showed CECDAI < 7.1, while 17 had CECDAI ≥ 7.1. All cases with PCDAI ≥ 10 also had CECDAI ≥ 7.1. Fisher’s exact test showed significant differences when categorized as PCDAI < 10 or CECDAI < 7.1 (Table 2). Group comparisons among MR (PCDAI < 10, CECDAI < 7.1), CR (PCDAI < 10, CECDAI ≥ 7.1), and flare (PCDAI ≥ 10, CECDAI ≥ 7.1) revealed significant differences in CRP and ESR, but not in LRG (Table 3). The Bonferroni test revealed no significant differences between the MR and CR groups. In contrast, significant differences were found between MR and flare for CRP (p < 0.001) and ESR (p < 0.001) and between CR and flare for ESR (p = 0.019).

**Table 2:** Relationship between SBCE findings and PCDAI Patients were categorized into four groups based on the combination of PCDAI < 10 and mucosal remission on SBCE, defined as LS < 135 and CECDAI < 7.1. Statistical analyses were performed using Fisher’s exact test. A significant association was observed between the PCDAI and LS (p = 0.02) and between the PCDAI and CECDAI (p < 0.001). SBCE: small-bowel capsule endoscopy; PCDAI: Pediatric Crohn’s Disease Activity Index; LS: Lewis score; CECDAI: Capsule Endoscopy Crohn’s Disease Activity Index.

		PCDAI		P value
		< 10	≥ 10	
LS	< 135	8	0	0.02
	≥ 135	24	21	
CECDAI	< 7.1	15	0	< 0.001
	≥ 7.1	17	21	

**Table 3:** Comparison of mucosal remission, clinical remission, and flare groups Data are expressed as a number (percentage) or median (interquartile range). Patients were categorized into three groups: (1) mucosal remission (PCDAI < 10 and LS < 135 or CECDAI < 7.1), (2) clinical remission (PCDAI < 10 and LS ≥ 135 or CECDAI ≥ 7.1), and (3) flare (PCDAI ≥ 10 and LS ≥ 135 or CECDAI ≥ 7.1). Demographics, biomarkers, and the number of LS segments with villous edema plus ulceration subscores ≥ 135 were compared across groups. LS: Lewis score; CECDAI: Capsule Endoscopy Crohn’s Disease Activity Index; SBCE: small-bowel capsule endoscopy; PCDAI:  Pediatric Crohn’s Disease Activity Index.

	Mucosal Remission	Clinical Remission	Flare	P value
LS				
Number	8	24	21	
Sex(male/female)	5/3 (63%/37%)	18/6 (75%/25%)	11/10 (52%/48%)	0.28
Age at SBCE[median(IQR)](years)	13.5 (12 - 15)	13 (12 - 15)	14 (13 - 16)	0.2
Disease duration[median(IQR)](month)	26 (8 - 44)	26 (7 - 50)	10 (5 - 32)	0.21
CRP[median(IQR)](mg/L)	0.1 (0.1 - 0.1)	0.5 (0.3 - 1.7)	3.0 (0.9 - 9.0)	< 0.001
ESR[median(IQR)](mm/h)	4 (2 - 6)	6 (3 - 9)	15 (10 - 28)	< 0.001
LRG[median(IQR)](µg/mL)	10.0 (9.4 - 10.7)	18.9 (17.4 - 20.5)	26.0 (18.2 - 30.2)	0.081
LS ≥ 135 Segments[median(IQR)]	0 (0 - 0)	2 (1 - 3)	3 (2 - 3)	< 0.001
CECDAI				
Number	15	17	21	
Sex(male/female)	10/5 (67%/33%)	13/4 (76%/24%)	11/10 (52%/48%)	0.32
Age at SBCE[median(IQR)](years)	13 (12.5 - 16)	12 (11 - 15)	14 (13 - 16)	0.068
Disease duration[median(IQR)](month)	41 (10 - 57)	15 (7 - 32)	10 (5 - 32)	0.098
CRP[median(IQR)](mg/L)	0.1 (0.1 - 0.5)	0.5 (0.3 - 1.6)	3.0 (0.9 - 9.0)	< 0.001
ESR[median(IQR)](mm/h)	3 (2 - 7)	8 (4 - 9)	15 (10 - 28)	< 0.001
LRG[median(IQR)](µg/mL)	10.0 (9.4 - 10.7)	18.9 (17.4 - 20.5)	26.0 (18.2 - 30.2)	0.081

**Figure 3:**
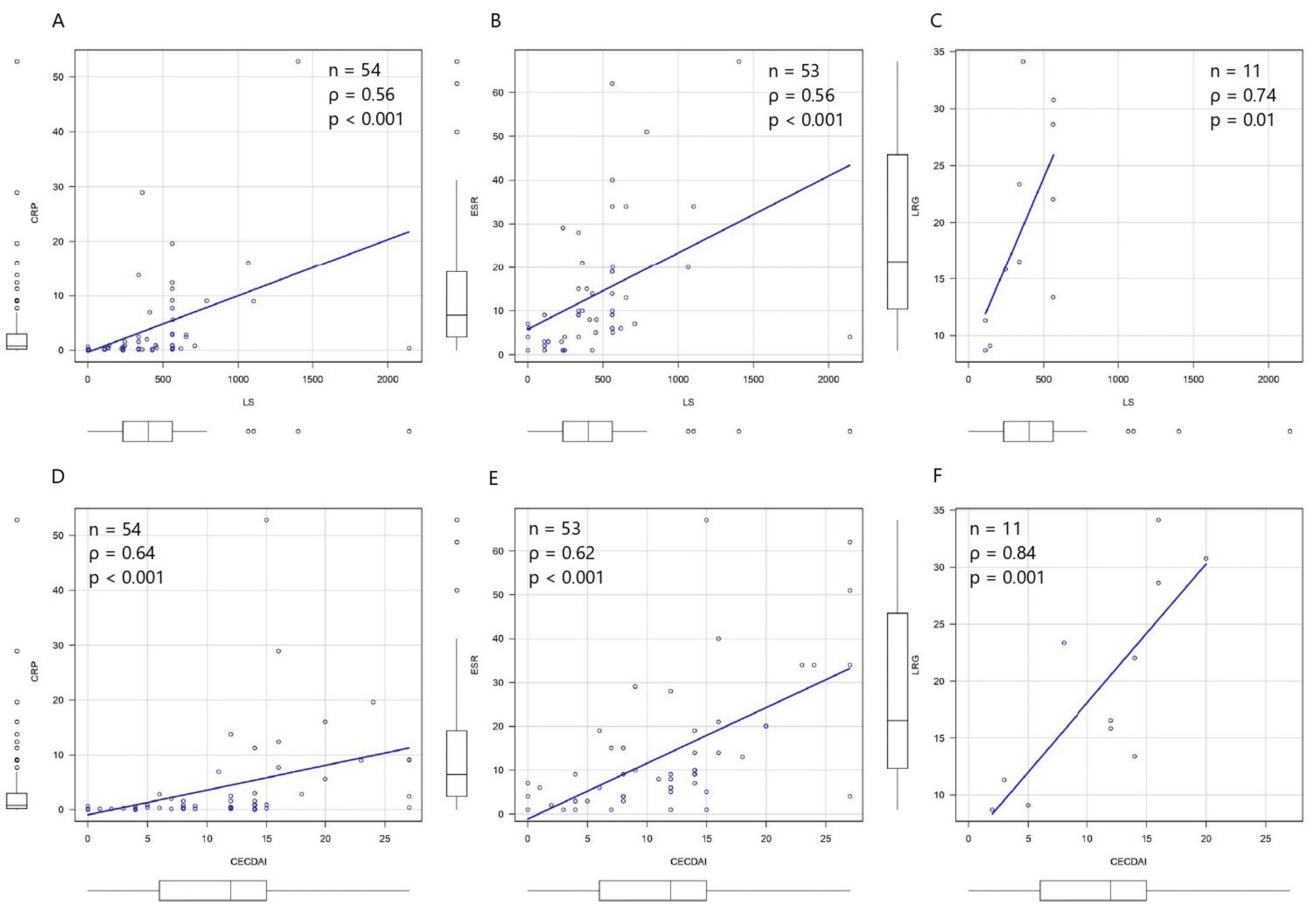
Correlation between biomarkers and SBCE findings Spearman’s rank correlation was used to assess the relationship between the biomarkers and SBCE scores. (A) CRP (n = 54) showed a positive correlation with LS (ρ = 0.56, p < 0.001) and (D) CECDAI (ρ = 0.64, p < 0.001). (B) ESR (n = 53) was also positively correlated with LS (ρ = 0.56, p < 0.001) and (E) CECDAI (ρ = 0.62, p < 0.001). (C) LRG (n = 11) showed the strongest correlations with LS (ρ = 0.74, p = 0.01) and (F) CECDAI (ρ = 0.84, p = 0.001). SBCE: small bowel capsule endoscopy; LS: Lewis score; CECDAI: Capsule Endoscopy Crohn’s Disease Activity Index.

LS was positively related to CRP, ESR, and LRG, and the association was most pronounced with LRG (ρ = 0.74, p = 0.01) (Figures 3A, 3B, 3C). ROC analysis for predicting LS < 135 identified cutoff values of 0.3 mg/L for CRP, 8 mm/h for ESR, and 13.4 µg/mL for LRG. Of the three biomarkers, LRG provided the most accurate discrimination, achieving 100% sensitivity and 88.9% specificity (Table 4).CECDAI was also positively related to CRP, ESR, and LRG, and the association was most pronounced with LRG (ρ = 0.84, p = 0.001) (Figures 3D, 3E, 3F). ROC analysis for predicting CECDAI < 7.1 identified cutoff values of 0.8 mg/L for CRP, 8 mm/h for ESR, and 13.4 µg/mL for LRG. Of the three biomarkers, LRG provided the most accurate discrimination, achieving 100% sensitivity and 100% specificity (Table 4).

**Table 4:** Diagnostic accuracy of biomarkers for small bowel mucosal remission according to LS and CECDAI ROC curve analyses were performed to determine optimal cut-off values for CRP, ESR, and LRG. Sensitivity, specificity, and AUC with 95% CI are summarized. ROC: receiver operating characteristic; LS: Lewis score; CECDAI: Capsule Endoscopy Crohn’s Disease Activity Index; AUC: area under the ROC curve; CI: confidence intervals.

Biomarker	Cut-off value	Sensitivity	Specificity	AUC	95% CI
	LS < 135				
CRP	0.3 mg/L	0.875	0.826	0.868	0.765 – 0.971
ESR	8 mm/h	0.875	0.644	0.801	0.668 – 0.935
LRG	13.4 µg/mL	1.000	0.889	0.944	0.790 – 1.000
	CECDAI < 7.1				
CRP	0.8 mg/L	0.812	0.658	0.791	0.668 – 0.915
ESR	8 mm/h	0.812	0.698	0.804	0.672 – 0.935
LRG	13.4 µg/mL	1.000	1.000	1.000	1.000 – 1.000

## Discussion

In this study, LS and CECDAI demonstrated a strong positive correlation in pediatric patients with CD (ρ = 0.82, p < 0.001). Previous reports in adult CD populations have similarly shown a strong correlation between these two scores (ρ = 0.81 - 0.88) [11,13]. Oliva et al. evaluated 312 pediatric CD patients and reported a significant correlation between LS and CECDAI (r = 0.772, p < 0.001) [18]. Importantly, data directly comparing LS and CECDAI in pediatric patients remains limited, despite the increasing use of SBCE in children. Our findings, therefore, provide valuable evidence supporting the consistency of these scoring systems in the pediatric population, suggesting that both scores can reliably reflect small-bowel inflammatory activity in clinical practice.

In adult CD patients, LS 135 corresponds to CECDAI 4.9 - 7.7, and LS 790 corresponds to CECDAI 6.9 - 10.3 [11-13]. In contrast, Arcos-Machancoses et al. reported that in pediatric patients with CD, LS 135 and 790 corresponded to CECDAI values of three and six, respectively [19]. In the present study, the CECDAI value was 7.1, comparable to previously reported values in adults; however, the CECDAI value equivalent to LS 790 was 16.5, higher than previously reported. This discrepancy may be attributed to the higher weight assigned to the strictures in the LS calculation. Furthermore, in a report by Arcos-Machancoses et al., 47.5% of patients had LS < 135, suggesting that the lower level of inflammation in their cohort may have influenced their results.

Both LS and CECDAI showed positive correlations with PCDAI (ρ = 0.47 and ρ = 0.57, respectively; p < 0.001), although a number of CR cases still showed active inflammation on SBCE. Turner et al. reported a correlation between PCDAI and the Simple Endoscopic Score for Crohn’s Disease (SES-CD) (ρ = 0.45, p < 0.001), but PCDAI < 10 had only 63% sensitivity and 77% specificity for predicting MR, indicating its limited utility [20]. Our findings are consistent with those of Turner et al., highlighting the insufficiency of symptom-based assessments alone in determining MR in long-term management. This supports incorporating SBCE-based objective assessment even in clinically quiescent patients to avoid underestimating residual inflammation.

In comparing LS and CECDAI, LS appeared to identify inflammatory changes during CR somewhat more frequently, as all eight cases with PCDAI < 10 and LS < 135 were included within the 15 cases with PCDAI < 10 and CECDAI < 7.1, whereas all 21 cases with PCDAI ≥ 10 and LS ≥ 135 corresponded exactly to those with PCDAI ≥ 10 and CECDAI ≥ 7.1. The LS uses the segment with the highest inflammatory score, while the CECDAI scores both halves of the SBTT and the sum of the scores. This difference may explain why LS detected localized inflammation, which CECDAI did not, particularly in CR cases. In flare cases with diffuse inflammation, the discrepancy between LS and CECDAI was less pronounced. In our cohort, the median number of segments with villous edema plus ulceration ≥ 135 in the PCDAI ≥ 10 and LS ≥ 135 group was three, indicating diffuse inflammation. Therefore, the LS may be more suitable for long-term assessments during CR. Few studies have compared the LS and CECDAI in pediatric patients with CD. In adults, Omori et al. suggested that the CECDAI may more accurately reflect active intestinal inflammation. In a study of 184 adult patients with CD, 30.9% showed discordance between LS and CECDAI, with 25.5% having inflammation only according to CECDAI and 5.4% according to LS alone [11]. Our study differs from theirs in that it focused on pediatric patients and did not include cases of strictures.

Several scoring systems for SBCE in pediatric CD have been reported, including the LS, CECDAI, and the Capsule Endoscopy - Crohn’s Disease (CE-CD) [8,9,18]. CE-CD is a novel scoring system for CD patients in which four endoscopic variables-number of ulcers, size of ulcers, proportion of the surface showing any signs of inflammation, and stenosis-are evaluated. The total CE-CD score ranges from 0 to 34 and has been shown to correlate positively with LS and the CECDAI [18,19]. Moreover, in an ROC curve analysis for predicting treatment escalation in pediatric CD, CE-CD was reported to be more useful than LS or CECDAI (AUC: 72%, 65%, and 71%, respectively) [19]. Since LS is integrated into Medtronic software, and LS and CECDAI are widely used in adult patients, we evaluated disease activity using LS and CECDAI in this study. Further accumulation of more cases is necessary to determine an optimal scoring system.

In this study, all biomarkers (CRP, ESR, and LRG) were positively correlated with SBCE scores, with LRG showing the strongest correlation. Several studies in adults have evaluated the association between small bowel inflammation and biomarkers [1,12-15,21-23]. They have reported a limited correlation or low sensitivity of CRP [1,12,13,21]. FC correlates positively with SBCE findings [12,22], and Selecting Therapeutic Targets in Inflammatory Bowel Disease II (STRIDE-II) recommends FC < 150 µg/g as a cutoff for mucosal healing [1]. Ukashi et al. noted that FC is more reflective of colonic inflammation than small bowel inflammation and that cutoff values for FC may vary by lesion location; they found that proximal small bowel inflammation was poorly reflected by FC, with a cutoff of 77 µg/g for mild proximal lesions [23]. Kawamoto et al. proposed an LRG cutoff of 13.4 µg/mL to predict small bowel ulcerative lesions identified by balloon-assisted enteroscopy, with 79% sensitivity and 82% specificity [14]. Another study showed positive correlations between LRG and both LS and CECDAI, with significantly lower LRG levels in patients achieving LS < 135 and CECDAI < 3.5 compared to those who did not (10.0 µg/mL vs. 15.2 µg/mL, p = 0.003) [15]. That study also suggested that combining colonic mucosal healing with LRG < 13.4 µg/mL may predict small bowel mucosal healing. The LRG cutoff in our study was consistent with Kawamoto’s, and it may be reasonable to defer endoscopic examination in cases with LRG < 13.4 µg/mL, where mucosal healing is likely.

This study had several limitations. Our study was retrospective in nature and had a small sample size. In particular, the number of LRG measurements was limited (n = 11), which may reduce the statistical power and generalizability of findings. Consequently, the high predictive performance reported for LRG may reflect overfitting, and these results should be interpreted with caution. Furthermore, some patients underwent multiple SBCE examinations during the study period, which may have introduced within-patient correlation and limited the generalizability of the findings. Neither colonoscopy nor esophagogastroduodenoscopy was performed simultaneously with the SBCE. Therefore, the influence of lesions other than those in the small intestine on the clinical symptoms and biomarkers cannot be completely ruled out. In addition, although an LS < 135 is commonly used as an indicator of MR in SBCE, this threshold remains arbitrary and has not been formally endorsed by STRIDE-II for the evaluation of small-bowel disease activity [1]. This limitation should be considered when interpreting the findings.

## Conclusions

This study confirmed that both LS and CECDAI are well-correlated and useful for assessing small bowel inflammation in pediatric Crohn’s disease. Our findings suggest that LS may have greater sensitivity for detecting subtle or subclinical small bowel inflammation in patients in CR. Furthermore, we identified LRG as a promising biomarker, with a level of less than 13.4 µg/mL strongly predicting MR. This suggests that combining LRG testing with clinical scores could help guide decisions on when to perform SBCE, thereby improving the efficiency of patient management. Future prospective, multicenter studies with larger cohorts are warranted to validate these findings and to establish optimized biomarker-based algorithms for determining the timing of SBCE in clinical practice.
